# Early apoptosis of porcine alveolar macrophages limits avian influenza virus replication and pro-inflammatory dysregulation

**DOI:** 10.1038/srep17999

**Published:** 2015-12-08

**Authors:** Pengxiang Chang, Suresh V. Kuchipudi, Kenneth H. Mellits, Sujith Sebastian, Joe James, Jinhua Liu, Holly Shelton, Kin-Chow Chang

**Affiliations:** 1 School of Veterinary Medicine and Science, University of Nottingham, Sutton Bonington, LE12 5RD, UK; 2 Veterinary and Biochemical Sciences, Pennsylvania State University, Wiley Lane, PA 16802, USA; 3 School of Biosciences, University of Nottingham, Sutton Bonington, LE12 5RD, UK; 4 Avian Infectious Diseases, The Pirbright Institute, Compton Laboratory, RG20 7NN, UK; 5 Key Laboratory of Animal Epidemiology and Zoonosis, Ministry of Agriculture, College of Veterinary Medicine and State Key Laboratory of Agrobiotechnology, China Agricultural University, Beijing, China

## Abstract

Pigs are evidently more resistant to avian than swine influenza A viruses, mediated in part through frontline epithelial cells and alveolar macrophages (AM). Although porcine AM (PAM) are crucial in influenza virus control, their mode of control is unclear. To gain insight into the possible role of PAM in the mediation of avian influenza virus resistance, we compared the host effects and replication of two avian (H2N3 and H6N1) and three mammalian (swine H1N1, human H1N1 and pandemic H1N1) influenza viruses in PAM. We found that PAM were readily susceptible to initial infection with all five avian and mammalian influenza viruses but only avian viruses caused early and extensive apoptosis (by 6 h of infection) resulting in reduced virus progeny and moderated pro-inflammation. Full length viral PB1-F2 present only in avian influenza viruses is a virulence factor that targets AM for mitochondrial-associated apoptotic cell death. With the use of reverse genetics on an avian H5N1 virus, we found that full length PB1-F2 contributed to increased apoptosis and pro-inflammation but not to reduced virus replication. Taken together, we propose that early apoptosis of PAM limits the spread of avian influenza viruses and that PB1-F2 could play a contributory role in the process.

Influenza A viruses are a major global health threat to humans and a wide range of susceptible animals. Pigs are widely regarded as “mixing vessels” for avian and mammalian influenza viruses contributing to the evolution of epidemic and pandemic viruses^1^,^2^. Typical swine influenza virus infections are acute and highly contagious, characterised by pyrexia, coughing, lethargy, weight loss, nasal discharge and dyspnoea^3^,^4^. Provided there is no secondary infection, recovery usually occurs over 3 to 7 days from start of infection^3^. Avian influenza virus infections in pigs, on the other hand, appear to be clinically mild compared with swine influenza virus infections. A comparative study of low pathogenicity avian influenza (LPAI) H5N2 virus and swine H1N1 virus infections in pigs found that the avian virus caused no clinical signs and produced lower virus titres than swine H1N1 virus infected pigs^4^. In another pig infection study with two swine viruses (H3N2 and H1N1) and four highly pathogenic avian influenza (HPAI) H5N1 viruses, titres of swine viruses from nasal swabs were much higher than those derived from H5N1 viruses^5^. The two swine viruses, likewise, caused more severe pathology and clinical signs than the H5N1 viruses^5^,^6^. Interestingly, lungs from swine virus infected pigs showed little evidence of apoptosis, as determined by TUNEL staining, in contrast to frequent detection of apoptotic cells in lungs of H5N1 virus infected pigs^5^. Pigs experimentally infected with HPAI H5N1 viruses also showed no transmission to in-contact pigs^6^,^7^. Furthermore, miniature pigs infected with the emergent avian H7N9 virus produced little or no clinical signs, in contrast to the severity of H7N9 virus infection in humans^8^. Taken together, pigs appear to be more clinically resistant to avian than swine influenza viruses, and correspondingly produce less virus progeny.

The initial site of influenza virus infection in the pig, as in human, is the respiratory tract where the first cell types to encounter the invading virus are respiratory epithelial cells and alveolar macrophages (AM); the latter are phagocytic cells externally sited on epithelial cells. AM are mainly found in the alveolar region of the lower respiratory tract where they readily migrate from adjacent capillaries. AM are essential in the control of influenza virus infection; their depletion in pigs, ferrets and mice resulted in severe infection and death^9^,^10^,^11^. Depletion of AM (with the use of dichloromethylene diphosphonate as liposome encapsulated clodronate) in lungs of pigs resulted in severe clinical signs and 40% mortality from human H1N1 virus infection^9^. Infected control (no AM depletion) pigs showed mild clinical signs and no mortality to the same virus. In infected pigs depleted of AM, tumour necrosis factor alpha (TNF-α) induction in lungs was significantly lower than those of infected control pigs. Induction of interleukin- (IL-) 10, a potent anti-inflammatory cytokine that inhibits the synthesis of TNF-α and granulocyte-macrophage colony-stimulating factor, was greater in infected AM-depleted pigs than corresponding infected controls^9^. Overall, AM depletion in pig lungs appears to severely dampen a protective pro-inflammatory response to virus infection. Similarly, depletion of AM in lungs of ferrets resulted in severe lung lesions, increased lung pro-inflammatory chemokines and viral titres, and 40% mortality from pandemic H1N1 virus infection. Inflammatory dysregulation from AM depletion appeared to have contributed to the lung damage. Infected control ferrets, on the other hand, showed mild clinical signs and no mortality to the same virus^10^.

Depletion of murine AM in lungs of C57BL/6 mice also resulted in severe pneumonia with dysregulated cytokine and chemokine production, and in 100% mortality from BJx-109 virus infection which was an engineered H3N2 virus (with PR8 [A/PR/8/34, H1N1] core proteins)^11^. BJx-109 virus was readily able to infect AM; most AM were viral NP positive 6–8 h post-infection as detected by immunofluorescence^11^. However, virus replication of BJx-109 virus in these murine AM was abortive i.e. there was no significant productive virus output. On the other hand, PR8 H1N1 virus infection in mice was severe and led to 100% mortality but the virus did not appear to infect AM. This murine study points to the role of abortive infection (BJx-109) in AM as an innate protective mechanism that limits progeny virus production, and in the absence of AM, or in their inability to be infected by influenza virus (such as PR8) the ensuing infection could be severe and fatal.

Given the ability of pigs to effectively resist LPAI and HPAI virus infections, and the functional importance of AM in the control of influenza virus infection we determined the relative resistance of PAM to infections with avian and mammalian influenza viruses in connection with their permissiveness to virus replication and host pro-inflammatory response.

## Results

### Avian but not swine or human influenza viruses caused extensive rapid damage to PAM

**Figure 1:**
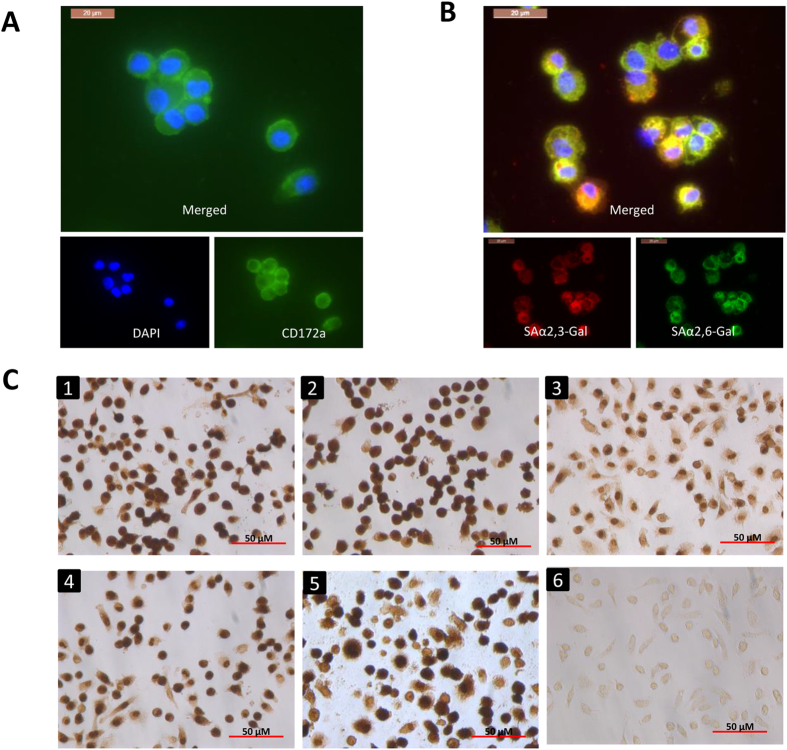
Avian and mammalian influenza viruses showed comparable early infectivity in PAM. CD172a (mouse anti-pig CD172a at 1:200 dilution, AbD Serotec) positive PAM as revealed by FITC conjugated secondary antibody (green) (**A**) co-expressed avian SAαa2,3-Gal (MAAII lectin binding) linked and human SAαa2,6-Gal (SNA lectin binding) linked receptors (**B**). Blue = DAPI nuclear detection. (**C**) Immunodetection of viral NP in PAM infected with (1) LPAI H2N3, (2) LPAI H6N1, (3) swine H1N1, (4) USSR H1N1, (5) pandemic H1N1 2009 at 1.0 MOI for 5 h or (6) no virus control showed that all viruses assessed were comparably infective in PAM.

**Figure 2:**
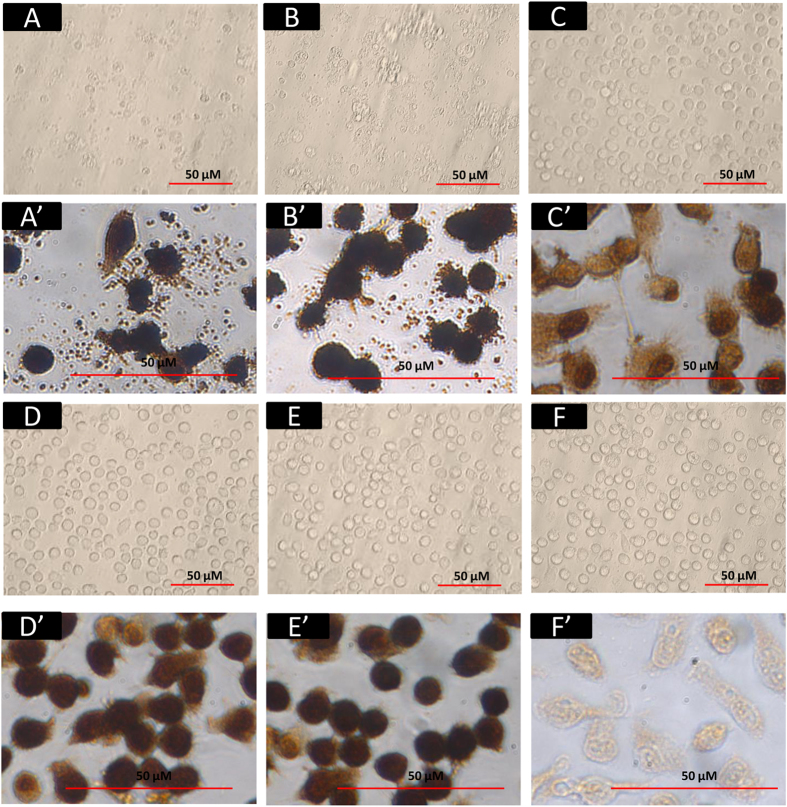
Avian but not mammalian influenza viruses caused severe damage to PAM. Avian viruses LPAI H2N3 (**A,A’**) and LPAI H6N1 (**B,B’**), and mammalian viruses swine H1N1 (**C,C’**), USSR H1N1 (**D,D’**) and pandemic H1N1 2009 (**E,E’**) were used at 1.0 MOI (based on focus forming units [FFU] titration on MDCK cells) to infect PAM for 6 h with mock infection as control (F and F’). (**A**–**F**) are bright field images and (**A’**–**F’**) are corresponding viral NP immunostained images. By 6 h of infection, widespread damage of PAM (cytopathogenicity, cell fragmentation and blebbings) was evident in PAM infected with avian influenza viruses ((**A**,**B**), (**A’**,**B’**)). By contrast, PAM infected with mammalian influenza viruses remained visibly normal ((**C**–**E**) and (**C’**–**E’**)), even beyond 24 h of infection (data not shown).

PAM from lung lavage of healthy 4- to 6-week-old pigs were positively immuno-stained with macrophage-specific antibody (mouse anti pig CD172a) and fluorescein isothiocyanate (FITC) conjugated secondary antibody (Fig. 1A)). Avian sialic acid α2,3-galactose (SAαa2,3-Gal) linked (MAAII lectin binding) and human SAαa2,6-Gal linked (SNA lectin binding) virus receptors were co-expressed in PAM (Fig. 1B). PAM were infected with two avian (low pathogenicity avian influenza [LPAI] A/mallard duck/England/7277/06 [H2N3] and LPAI A/turkey/England/198/09 [H6N1]), and three mammalian (A/swine/England/117316/1986 [swine H1N1], human A/USSR/77 [USSR H1N1] and human A/California/07/2009 [pandemic H1N1 2009]) influenza viruses for 5h at multiplicity of infection (MOI) of 1.0, based on virus titrations (focus forming assays) on Madin Darby canine kidney (MDCK) cells. PAM showed comparable susceptibility to early infection to all five viruses in that all PAM were similarly positive for viral nucleoprotein (NP) (Fig. 1C). However, by 6 h of infection, extensive cell damage (cellular fragmentation and blebbing), indicative of acute apoptosis, was evident in avian (Fig. 2A,A’) but not mammalian influenza virus infected PAM (Fig. 2C,C’–E,E’). Mammalian influenza virus infected PAM remained phenotypically normal throughout culture (≥24h). This finding suggests that the severe damage of PAM in response only to the two avian influenza viruses could be a porcine specific innate immune response.

### Avian influenza virus infected PAM displayed accelerated apoptosis and reduced progeny virus release

**Figure 3:**
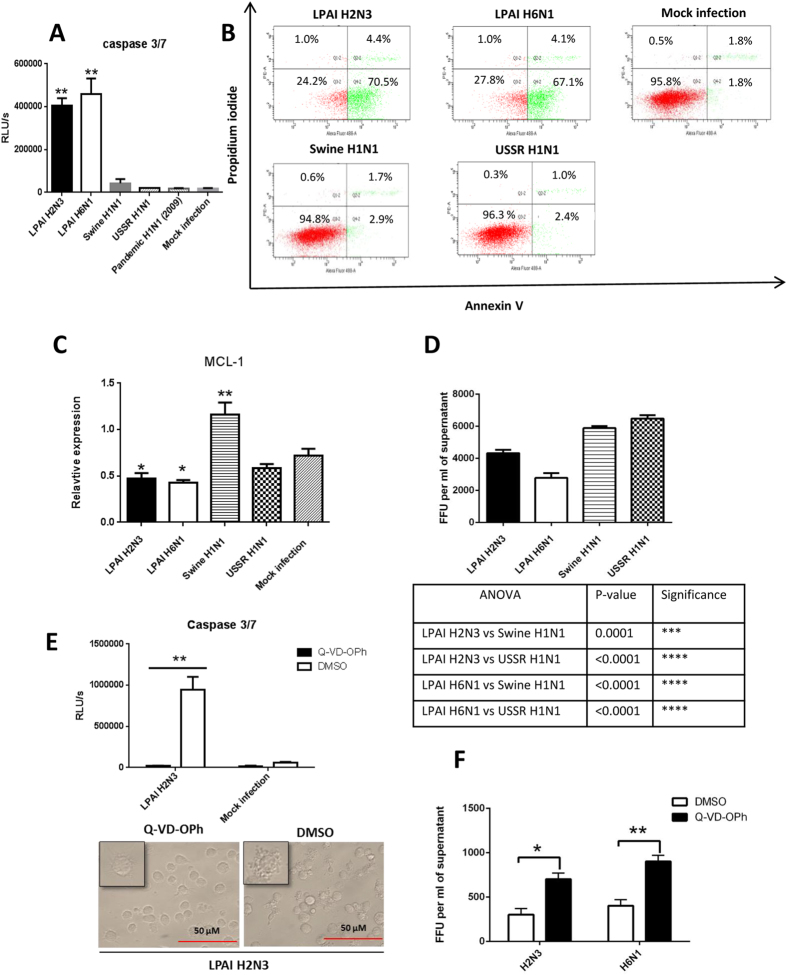
Avian influenza virus infected PAM displayed rapid apoptosis and reduced progeny virus release. PAM were infected with avian and mammalian viruses as indicated at MOI of 1.0 for 6 h to determine host responses in (**A**) caspase 3/7 activation, (**B**) relative presentation of annexin V and uptake of propidium iodide (PI), (C) messenger RNA expression of *Mcl1*, and (**D**) infectious virus release into culture supernatants. PAM infected with avian influenza viruses showed (**A**) much higher caspase3/7 activation, and (**B**) more detectable annexin V with little evidence of necrosis as indicated by lack of PI uptake. (**C**) Marked apoptosis detected in avian virus infected PAM coincided with the down-regulation of *Mcl1* (expression normalized to 18S rRNA) by avian H2N3 and H6N1 viruses. (**D**) Less avian than mammalian progeny virus was released from 6 h infected PAM, as determined by virus titration on MDCK cells with culture supernatants. (**E**,**F**) PAM were infected with LPAI H2N3 or LPAI H6N1 virus at 1.0 MOI; after 1 h of initial virus incubation the cells were washed with PBS and incubated in fresh infection culture media with 20 μM Q-VD-OPh, a pan-caspase inhibitor, for a further 5 h. (**E**) Infected PAM in the presence Q-VD-OPh showed no caspase 3/7 activation and no apparent cytopathic or apoptotic damage, unlike infected DMSO treated PAM. (**F**) Q-VD-OPh inhibition of apoptosis in PAM increased viable progeny release of H2N3 and H6N1 viruses. Data shown are the means of triplicate wells. Error bar = standard error of mean. *P < 0.05, **P < 0.001 (ANOVA with the use of Prism [GraphPad]).

PAM infected with avian, but not mammalian, influenza viruses (at MOI of 1.0 for 6 h) showed marked caspase3/7 activation (Fig. 3A), extensive annexin V binding to phosphatidylserine on cell membrane (Fig. 3B) and down-regulation of *myeloid cell leukemia 1* (*Mcl1*), a potent inhibitor of apoptosis in macrophages^12^ (Fig. 3C), all of which indicated the induction of apoptosis. Rapid down-regulation of Mcl1, a membrane-bound Bcl2 family protein, is known to promote the initiation of the apoptotic cascade^13^,^14^. PAM infected with avian influenza viruses also produced less infectious progeny virus than those infected with mammalian viruses (Fig. 3D). Treatment of PAM with Q-VD-OPh, a pan-caspase inhibitor, blocked caspase 3/7 activation and preserved morphological integrity in the presence of avian H2N3 virus (Fig. 3E) which also correlated with increased viable output of H2N3 and H6N1 viruses (Fig. 3F).

### Avian influenza viruses appeared more pro-inflammatory than swine or human influenza viruses in PAM

**Figure 4:**
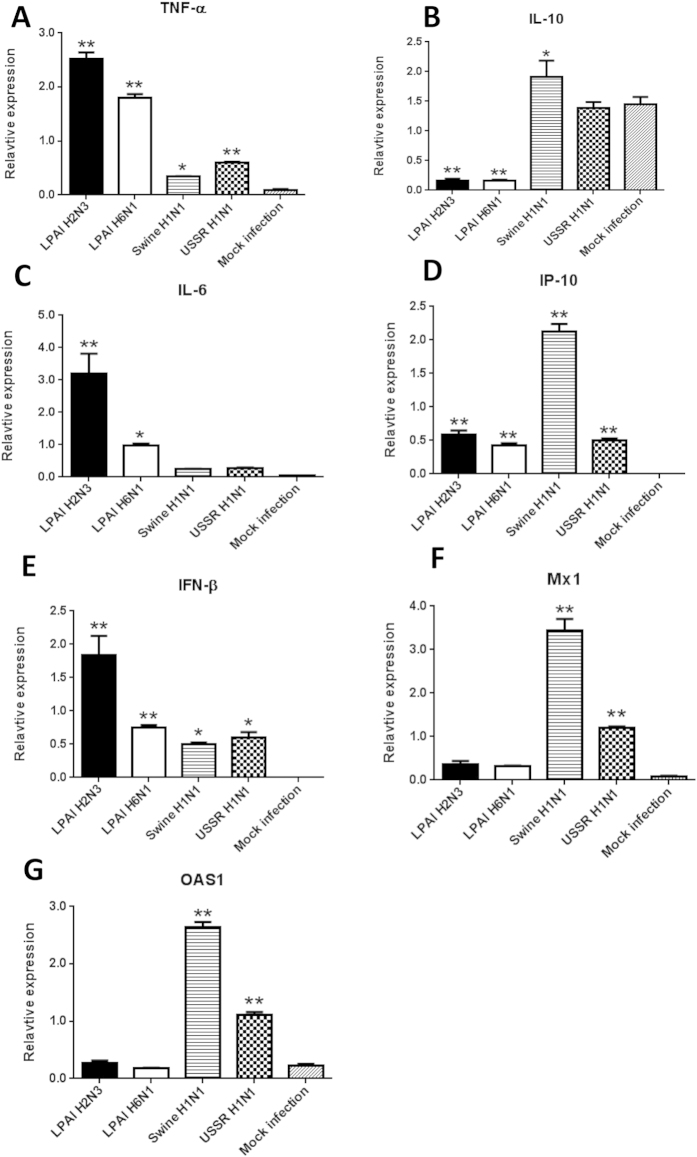
Avian influenza virus infected PAM appeared more pro-inflammatory than those infected with human and pig influenza viruses. PAM were infected with influenza viruses as indicated at 1.0 MOI for 6 h. PAM infected with avian influenza viruses exhibited more *TNF-α* and *IL-6* transcription than with mammalian influenza viruses (**A**,**C**). Conversely, *IL10* expression in PAM was strongly down-regulated by avian but not mammalian influenza viruses (**B**). There was no clear difference in the induction of *IP-10* (**D**) and *IFN-β* (**E**) in PAM between avian and mammalian influenza viruses. Mammalian influenza viruses, however, strongly up-regulated the expression of IFN-inducible genes *Mx1* and *OAS1* (**F**,**G**). All RNA expression results were normalised to 18S rRNA. Data shown are the means of triplicate wells. Error bar = standard error of mean. *P < 0.05, **P < 0.001 (ANOVA).

Besides the task of virus removal, AM are expected to play key roles in the establishment of early pro-inflammatory response to influenza virus infection which allows the attraction of other key cellular responders of the immune response. PAM at 6 h infection expressed *TNF-α* and *IL-6* (pro-inflammatory cytokines) more highly with the two avian than the three mammalian influenza viruses (Fig. 4A,C). Conversely, *IL10* (anti-inflammatory cytokine) expression in PAM was strongly down-regulated by the avian but not the mammalian influenza viruses (Fig. 4B). This inverse expression relationship of up-regulated *TNF-α* and down-regulated *IL-10* in PAM infected with avian influenza viruses is similar to the expression profile of AM from pigs challenged with a human H1N1 virus where the infected pigs only showed mild clinical signs^9^. In that same study, infected pigs, chemically depleted of AM, showed severe clinical signs that coincided with reduced *TNF-α* and highly induced *IL-10* expressions. The limited induction of *TNF-α* and *IL-6* together with sustained *IL-10* expression in mammalian influenza virus infected PAM could indicate a sub-optimal pro-inflammatory response (Fig. 4A–C). Speculatively, elevated TNF-α and reduced IL-10 in avian influenza virus infected PAM could indicate a more balanced paracrine/endocrine pro-inflammatory signature during infection.

There was no clear difference between avian and mammalian influenza viruses in the induction of *IP-10* and *IFN-β* in PAM (Fig. 4D,E). However, mammalian influenza viruses strongly up-regulated the expression of *Mx1* and *OAS1* (Fig. 4F,G) which was likely to be a reflection of the presence of viable but infected cells at 6 h of infection.

### Mutations of PB1-F2 in recombinant avian H5N1 virus reduced apoptosis and dampened pro-inflammatory response in PAM

Most (96%) influenza viruses isolated from avian hosts harbour full length (87-90 amino acids) PB1-F2 proteins^15^,^16^. The sizes of PB1-F2 in avian H2N3, avian H6N1, swine H1N1, human USSR H1N1 and pandemic H1N1 2009 viruses are 90, 90, 57, 52 and 11 amino acids in length respectively, as determined by sequencing (Table 1). PB1-F2 of influenza virus has been shown to be a virulence factor that targets AM for mitochondrial-associated apoptotic cell death^4^ but this is strain specific^1^,^13^. Induction of inflammation, on the other hand, is a more consistent feature of full length PB1-F2^17^.

**Figure 5:**
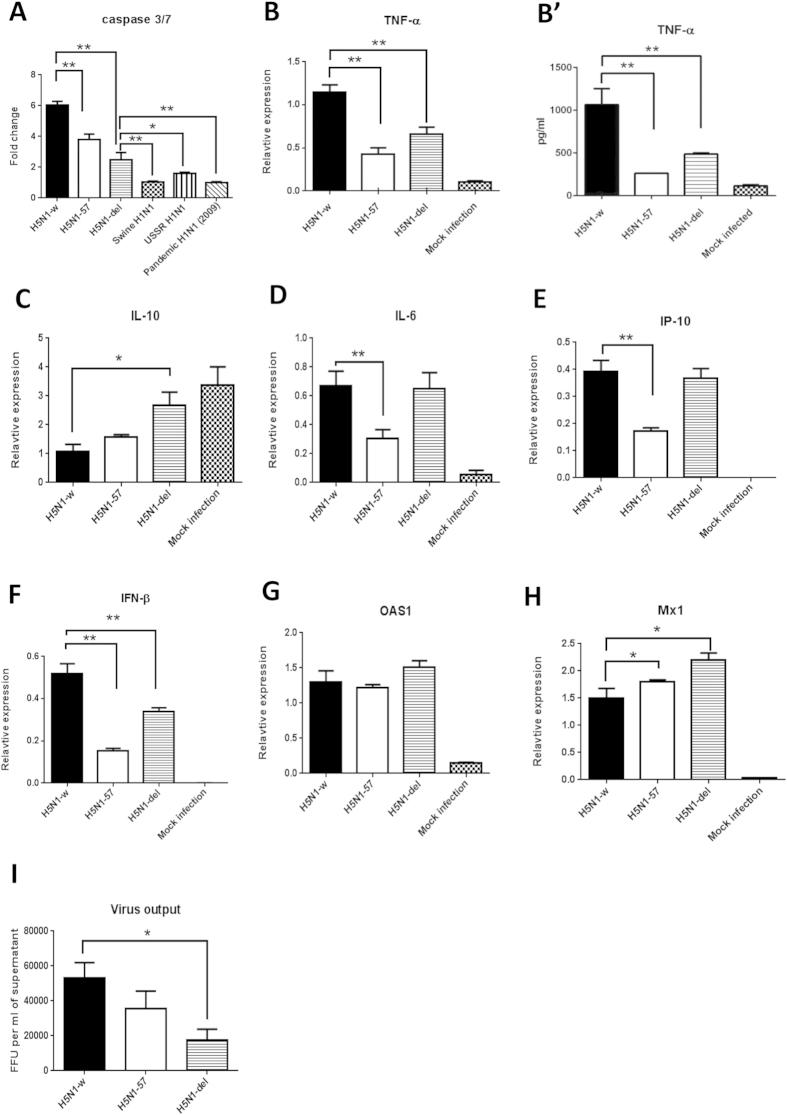
Contribution of viral PB1-F2 protein to avian influenza promotion of apoptosis and pro-inflammation in PAM. Recombinant H5N1 (H5N1-w, H5N1-57 and H5N1-del) and mammalian influenza viruses, as indicated, were used to infect PAM at MOI of 1.0 for 6 h. (**A**) Both truncated (H5N1-57) and deleted (H5N1-del) PB1-F2 mutants showed significantly reduced activation of caspase 3/7 in PAM relative to the wild type virus (H5N1-w) but the levels were not down to the same basal levels as those from mammalian viruses. Fold changes were in relation to mock control. (**B**,**B’**) The two PB1-F2 mutants conferred reduced *TNF-α* induction of RNA (**B**) and protein (**B’**) in PAM compared with the wild type virus. (**C**) H5N1-del mutant also maintained elevated expression of *IL-10* in PAM, unlike that of the wild type which suppressed *1L-10* expression. Hence PB1-F2 mutations in H5N1-w virus appeared to dampen apoptotic and pro-inflammatory responses. (**D**–**F**) The induction of *IL-6*, *IP-10* and *IFN-β* by the three recombinant H5N1 viruses, however, followed a less clear-cut pattern. (**E**) Induction of *IL-6* and *IP-10* was weakest with the truncated PB1-F2 mutant (H5N1-57), and was similarly high between the deleted mutant (H5N1-del) and wild type virus. H5N1-w (wild type) virus induced the strongest *IFN-β* response (**F**) but the induction of *OAS1* (**G**) and *Mx1* (**H**) did not follow the same trend. (**I**) PB1-F2 mutant viruses in PAM produced less progeny virus than the parental wild type. Data shown are the means of triplicate wells. Error bar = standard error of mean. *P < 0.05, **P < 0.001 (ANOVA).

We evaluated the possible contribution of a full length PB1-F2 (90 amino acids) in a reverse genetics engineered avian H5N1 (H5N1-w) virus to promote apoptosis and pro-inflammation in PAM. A reverse genetics system for H5N1-w, comprising HA and NA from PR8 H1N1 virus (A/Puerto-Rico/8/34) and core genes from HPAI H5N1 virus (A/turkey/England/50-92/91)^18^ that allowed experimentation under containment level 2 conditions, was used to generate a truncated (H5N1-57) and a deleted (H5N1-del) PB1-F2 mutant virus. Both PB1-F2 mutant viruses did not alter the coding of PB1 nor expression levels of PB1 or PB1-N40 (data not shown). Both H5N1-57 and H5N1-del viruses showed significantly reduced caspase 3/7 activation in PAM relative to the H5N1-w virus but the levels were not down to the same basal levels as those from mammalian viruses (Fig. 5A). The two PB1-F2 mutants conferred reduced *TNF-α* induction in PAM compared with the wild type (Fig. 5B). The H5N1-del mutant also maintained elevated expression of *IL-10* in PAM, unlike that of the wild type which suppressed *1L-10* expression (Fig. 5C). The induction of *IL-6*, *IP-10* and *IFN-β* (Fig. 5D–F) by the three recombinant H5N1 viruses, however, followed a less clear-cut pattern. Induction of *IL-6* and *IP-10* was weakest with the truncated PB1-F2 mutant (H5N1-57) (Fig. 5D,E). H5N1-w (wild type) virus induced the strongest *IFN-β* response (Fig. 5F) but the induction of *OAS1* and *Mx1* did not follow the same trend (Fig. 5G,H). Overall, PB1-F2 mutations in H5N1-w virus appeared able to dampen apoptotic and pro-inflammatory responses (Fig. 5A–C). Notably, PB1-F2 mutant viruses in PAM produced less progeny virus than the parental wild type (Fig. 5I). In summary, full length PB1-F2 appears to contribute to increased apoptosis and pro-inflammation but not to reduce virus proliferation in PAM.

Although other viral proteins (M1 and NS1) may also elicit apoptosis^19^, we identified in PAM a dramatic host apoptotic response to the presence of avian influenza viruses that resembles an earlier observation of extensive cell death of primary porcine respiratory epithelial cells from LPAI and HPAI virus infections which were not seen in corresponding primary human cells^20^. Taken together, we propose that early apoptosis of PAM is a protective host-species response that limits the spread of avian influenza viruses in pigs and that full length PB1-F2 contributes to apoptotic and pro-inflammatory responses.

## Discussion

We showed in the present study that PAM were permissive to initial infection with avian and mammalian influenza viruses (Fig 1). Extensive apoptosis in avian influenza virus infected PAM was evident by 6 h of infection (Fig. 2) with caspase activation (Fig. 3A,B) and reduced *Mcl1* expression (Fig. 3C). Mcl1 is a membrane-bound Bcl2 family protein which promotes cell survival by interfering at an early stage in the apoptotic cascade that leads to the release of cytochrome c from mitochondria^13^,^14^. It has a short half-life (typically one to a few hours), is rapidly down regulated by caspases during apoptosis and is an essential regulator of macrophage lifespan^12^. PAM infected with mammalian viruses, on the other hand, remained phenotypically normal well beyond 6 h of culture. In addition, avian virus infected PAM produced less progeny virus than PAM with mammalian viruses (Fig. 3D). Predictably, pan-caspase inhibition (with Q-VD-OPh) of apoptosis in PAM increased avian H2N3 and H6N1 virus output (Fig. 3E,F). Furthermore, PAM infected with avian influenza viruses appeared more pro-inflammatory than those infected with mammalian influenza viruses (Fig. 4).

Mechanistic insights into innate immune resistance to virulent influenza infection could enable the development of novel interventions for its treatment and control. AM and closely associated respiratory epithelial cells are cell types of major importance in the control and clearance of respiratory pathogens. They are the first cells to encounter incoming pathogens, and initiate the host innate immune response that will dictate the conduct of subsequent innate and acquired immune actions. Pigs are evidently more resistant to LPAI and HPAI virus infections than to swine and human influenza virus infections, manifested as reduced virus output and less vigorous pro-inflammatory response from both respiratory epithelial cells and peripheral blood monocyte-derived macrophages (PBMM)^3^,^4^,^5^,^20^. Primary pig tracheal epithelial cells infected with HPAI H5N1 virus displayed higher cell death than correspondingly infected primary human respiratory epithelial cells^20^ suggesting that the porcine cell response is analogous to infected primary duck cells which undergo rapid apoptosis to limit virus replication^21^. Interestingly, less cytokines and progeny virus were produced from human AM than peripheral blood monocyte-derived macrophages (PBMM) when infected with HPAI H5N1 viruses^22^,^23^, which suggests that AM are less prone to hypercytokinemic dysregulation than PBMM, and that PBMM are not good models for AM^22^.

The mild clinical features and low virus output of HPAI H5N1 virus infections in pigs coincided with frequent detection of apoptotic lung cells^5^. By contrast, swine influenza virus infections caused more severe pathology, clinical signs and higher virus output but little or no detection of apoptotic cells^5^. This observation in pigs that apoptosis in response to HPAI H5N1 virus infection favours host resistance is corroborated by our present *in vitro* finding of extensive and rapid apoptosis of PAM in response to avian influenza virus infections. Previous work on PAM infected with human H1N1 or H3N2 virus over five days found no evidence of apoptosis^24^, consistent with our findings that acute apoptosis was primarily seen in PAM infected with avian influenza viruses (Fig. 3). These *in vivo* observations and our present data suggest that rapid apoptosis in response to avian influenza infection in pigs could be a host protective response and that adaptation of viruses in swine has removed this response to make them more successful in this host. Previously, it was believed that infection of AM with influenza viruses was abortive resulting in no or insignificant release of viable virus progeny based on human and murine AM findings^11^,^25^. Abortive AM infection is not due to restricted virus entry but to post-entry inhibition^11^,^26^. However, it is becoming clear that certain HPAI H5N1 viruses associated with high virulence are able to undertake productive replication in human or murine AM^23^,^26^. Interestingly, in mice whose AM were unable to be infected with PR8 influenza virus, the ensuing systemic infection was fatal^11^. Collectively, PAM in the face of early avian influenza virus infection would appear to play a crucial role in limiting virus production and spread by phagocytosis of infected epithelial cells and by becoming targets of direct but limiting infection due to ensuing rapid apoptosis^11^,^19^,^26^. Interestingly, apoptosis of avian influenza virus-infected PAM was also accompanied by elevated expression of *TNF-α* and *IL-6*, and reduced *IL-10* expression relative to mammalian virus-infected PAM (Fig. 4) which is similar to the expression profile of AM from pigs that showed mild clinical signs in response to a human H1N1 virus challenge^9^. The ability of PAM to undertake early apoptosis and differentially express a favourable cytokine profile in response to avian influenza virus infection seems to be both a virus strain-specific and a host species-specific response.

Full length avian PB1-F2 appears to contribute to the rapid apoptosis and pro-inflammatory activation in PAM infected with avian influenza viruses. Truncated and deleted PB1-F2 mutant H5N1 viruses were less able to activate caspases 3/7 and conferred less TNF-α induction in PAM than wild type virus (H5N1-w) (Fig. 5A,B). However, given that PB1-F2 mutant viruses in PAM produced less progeny virus than the parental wild type, a full length avian PB1-F2 alone could not account for reduced virus proliferation seen with our avian viruses in comparison with mammalian viruses. Therefore, a full length PB1-F2 in avian influenza virus appears to contribute only in part to the phenomenon of rapid apoptosis and pro-inflammation in PAM.

A recent study examined the role of PB1-F2 of two swine H3N2 viruses in pigs^27^. The two viruses were triple assortants, each with a full length (90 amino acids) PB1-F2 of human origin. Wild type and null PB1-F2 of both swine viruses had no effect on virus shedding and virus accumulation in the lungs of infected pigs. PB1-F2 also showed no consistent effect on pulmonary cytokine induction. In the same study, PB1-F2 in both swine viruses did not produce consistent effects on PAM viability and progeny virus production; only the PB1-F2 null mutant of one H3N2 virus (sw/99 WT) generated less progeny virus than the wild type virus. There was, however, some reduction in the number of apoptotic PAM from both swine PB1-F2 null mutant viruses, in relation to corresponding wild type viruses^27^. In summary, the role of PB1-F2 in the two swine H3N2 viruses on virus replication and cell viability/apoptosis in PAM appears inconsistent and marginal. PAM survived well for at least several days with each swine H3N2 virus at high MOI (<10.0) regardless of the presence of PB1-F2^27^. This unremarkable response of PAM to swine H3N2 viruses is in sharp contrast to the response to avian influenza viruses where by 6 h of infection rapid and extensive apoptosis set in which suggests that full length PB1-F2 in a natural host (i.e. swine influenza virus in swine) does not trigger any major host effect. Other viral proteins (M1 and NS1) from different strains of human and avian influenza viruses have been shown to induce or inhibit apoptosis^19^. The ability of AM to undergo abortive avian influenza virus infection via early apoptosis, and phagocytise infected epithelial cells^28^ could be an important innate protective mechanism in limiting virus production and spread^11^,^26^. Although hemagglutinin subtype is a viral determinant of productive replication in AM^26^, host mechanisms responsible for abortive infections in AM are at present unclear.

## Methods

### Influenza viruses and cells

A LPAI H6N1 virus (A/turkey/England/198/09), a LPAI H2N3 virus (A/mallard duck/England/7277/06), a swine H1N1 (A/swine/England/117316/1986), a human pandemic H1N1 2009 (A/California/07/2009), a human USSR H1N1 (A/USSR/77 [USSR]), a recombinant highly pathogenic avian influenza H5N1-PR8 virus (A/turkey/England/50-92/91 with HA and NA from PR8 virus) referred as H5N1-w, a H5N1-PR8 with PB1-F2 truncated to 11 amino acids (H5N1-del), and a H5N1-PR8 with PB1-F2 truncated to 57 amino acid (H5N1-57) were used in this study. All the viruses were propagated in 10-day-old embryonated chicken eggs and allantoic fluid was harvested at 48 h post inoculation. Virus was aliquoted and stored in −80 °C until further use. PAM were collected by bronchoalveolar lavage and purified by Histopaque-1077 (Sigma) according to manufacturer's instructions. Cells were re-suspended in RPMI 1640 medium (Life Technologies) supplemented with 2 mM glutamine (Life Technologies), 100 U/ml penicillin streptomycin (Invitrogen), 1% non-essential amino acids (Invitrogen), and 1 mM sodium pyruvate (Invitrogen) and seeded in 6-well or 96-well plates (Sarstedt) for 1 h. Non-adherent cells were removed by washing twice with phosphate buffered saline (PBS) (Invitrogen). Bound cells were cultured in RPMI 1640 medium supplemented as above. MDCK cells were grown in DMEM-Glutamax I (high glucose) (Life Technologies) supplemented with 10% foetal calf serum and 100 U/ml penicillin streptomycin.

### Influenza receptor detection

Influenza receptor distribution on primary cells was determined by lectin cytochemical staining with FITC labelled *Sambucus nigra* agglutinin (SNA) lectin (Vector Labs) specific for human influenza receptor, sialic acid α2,6-galactose (SAα2,6-Gal), and biotinylated *Maackia amurensis* agglutinin II (MAA II) (Vector Labs) specific for avian influenza receptor type, sialic acid α2,3-galactose (SAα2,3-Gal) as described^29^,^30^.

### Infection and progeny virus quantification

PAM were infected with specific viruses at specified MOIs for 2 h in supplemented serum-free RPMI-1640 with 250 ng/ml TPCK trypsin (Sigma). After 2 h, cells were rinsed three times with PBS and incubated in fresh medium for a further 4 h. Quantification of infectious virus in PAM culture supernatants was conducted as previously described^21^ which was an immuno-cytochemical focus forming assay based on infection of MDCK cells followed by immunodetection of viral nucleoprotein (NP) expression. Briefly, MDCK cells infected for 6 h were fixed in acetone methanol for 10 min followed by peroxidase treatment for 10 min and incubation with a 1:8000 dilution of primary mouse monoclonal antibody to influenza nucleoprotein (Abcam) for 40 min at room temperature. The cells were subsequently rinsed with Tris-buffered saline (TBS), incubated with horse radish peroxidase-labelled polymer for 40 min. After gently rinsing with TBS, the cells were incubated with DAB substrate-chromogen solution for 7 min (Envision + system-HRP kit, Dako). Cells positive for viral nucleoprotein were counted with an inverted microscope and the mean of positive cells in four 96-wells was used to calculate infectious focus-forming units of virus per microlitre of infection volume.

### Caspase 3/7 assays and Q-VD-OPh caspase inhibition

PAM were treated with 20 μM Q-VD-OPh (Cambridge Bioscience), a pan-caspase inhibitor, or equivalent volume of DMSO (Sigma) for 3 h, washed twice with PBS and incubated with specified influenza virus at 1.0 MOI for 1 h. Infected PAM were then rinsed three times with PBS and incubated in fresh supplemented RPMI-1640 containing 20 μM Q-VD-OPh for a further 5 h. Activated caspase 3 and 7 in PAM were quantified using a Caspase-Glo 3/7 Assay (Promega) kit according to manufacturer's instructions.

### Apoptosis determination and TNF-α quantification

PAM apoptosis/necrosis was quantified using an Annexin V-FITC kit (Miltenyi Biotec) in a BD FACS CANTO II flow cytometer (BD Biosciences) and the results were analysed using FACSDiva software (BD Biosciences). Porcine TNF-α enzyme-linked immunosorbent assays (ELISAs) (Life Technologies) were performed with supernatants from PAM infected with 1.0 MOI of H5N1-w, H5N1-57 or H5N1-del virus for 6 h.

### RNA preparation and real-time RT-PCR

Total RNA was extracted from cells using an RNeasy Plus Minikit (Qiagen). cDNA was synthesized from 1 μg of total RNA using Superscript III First Strand synthesis kit (Invitrogen). Expression of host genes was performed with a LightCycler-480 instrument (Roche), using a relative standard curve approach, normalised to 18S rRNA. Sequence details of porcine *IL-10*: GCATCCACTTCCCAACCA (forward), CTTCCTCATCTTCATCGTCAT (reverse). Sequences of primers and probes of other porcine genes used are previously described^20^.

### Statistical analysis

Statistical analysis was performed using GraphPad Prism 6 (GraphPad Software). Paired student t test, one-way ANOVA and two-way ANOVA were used to test differences between different groups. P values <0.05 were considered significant.

## Additional Information

**How to cite this article**: Chang, P. *et al.* Early apoptosis of porcine alveolar macrophages limits avian influenza virus replication and pro-inflammatory dysregulation. *Sci. Rep.* **5**, 17999; doi: 10.1038/srep17999 (2015).

## Figures and Tables

**Table 1:** Length polymorphisms of PB1-F2.

Influenza viruses	Amino acids length of PB1-F2
HPAI H5N1 (A/turkey/England/50-92/91)	90
LPAI H2N3	90
LPAI H6N1	90
Pandemic H1N1 (2009)	11
USSR H1N1	57
Swine H1N1	52
